# Ecogenomics of microbial communities in bioremediation of chlorinated contaminated sites

**DOI:** 10.3389/fmicb.2012.00351

**Published:** 2012-10-02

**Authors:** Farai Maphosa, Shakti H. Lieten, Inez Dinkla, Alfons J. Stams, Hauke Smidt, Donna E. Fennell

**Affiliations:** ^1^Laboratory of Microbiology, Wageningen UniversityWageningen, Netherlands; ^2^Bioclear BVGroningen, Netherlands; ^3^Rutgers University, New BrunswickNJ, USA

**Keywords:** bioremediation, ecogenomics, transcriptomics, organohalide respiring bacteria, dehalococcoides, Dehalobacter, chloroethenes, reductive dehalogenase

## Abstract

Organohalide compounds such as chloroethenes, chloroethanes, and polychlorinated benzenes are among the most significant pollutants in the world. These compounds are often found in contamination plumes with other pollutants such as solvents, pesticides, and petroleum derivatives. Microbial bioremediation of contaminated sites, has become commonplace whereby key processes involved in bioremediation include anaerobic degradation and transformation of these organohalides by organohalide respiring bacteria and also via hydrolytic, oxygenic, and reductive mechanisms by aerobic bacteria. Microbial ecogenomics has enabled us to not only study the microbiology involved in these complex processes but also develop tools to better monitor and assess these sites during bioremediation. Microbial ecogenomics have capitalized on recent advances in high-throughput and -output genomics technologies in combination with microbial physiology studies to address these complex bioremediation problems at a system level. Advances in environmental metagenomics, transcriptomics, and proteomics have provided insights into key genes and their regulation in the environment. They have also given us clues into microbial community structures, dynamics, and functions at contaminated sites. These techniques have not only aided us in understanding the lifestyles of common organohalide respirers, for example *Dehalococcoides*, *Dehalobacter*, and *Desulfitobacterium*, but also provided insights into novel and yet uncultured microorganisms found in organohalide respiring consortia. In this paper, we look at how ecogenomic studies have aided us to understand the microbial structures and functions in response to environmental stimuli such as the presence of chlorinated pollutants.

## CHLORINATED SITES AND REMEDIATION CHALLENGES

For more than 100 years, a large variety of halogenated hydrocarbons have found widespread and massive application in industry, agriculture, and private households, for example, as biocides, solvents, degreasers, flame retardants, and in polymer production. This has resulted in the accidental and deliberate release of large quantities of these chemicals into the environment (Stringer and Johnston, 2001). On the other hand, over 4000 different halogenated hydrocarbons can be produced naturally by marine sponges, algae, bacteria, terrestrial plants, fungi, and insects, in many cases by eukaryote host-associated microbial communities (Gribble, 2003). Whereas in marine environments, mostly brominated compounds are produced, chlorinated metabolites dominate in terrestrial systems, however, it should be noted that in many cases, halogenases and dehalogenases known to produce/degrade chlorinated compounds are also active toward brominated equivalents. Bioremediation technology uses microorganisms to reduce, eliminate, or transform to benign products such halogenated contaminants present in soils, sediments, water, or air. Bioremediation technologies are increasingly being considered or applied for various types of contaminants, including solvents, explosives, polycyclic aromatic hydrocarbons (PAHs), polychlorinated biphenyls (PCBs), and polybrominated diphenyl ethers (PBDEs; Adrian et al., 2000; Futamata et al., 2007; Hiraishi, 2008; Cheng and He, 2009; Fennell et al., 2011). There is wide diversity of highly toxic and persistent organohalides in the environment, and implementation of bioremediation techniques is needed to remove them or render them harmless. The halogenated hydrocarbons with a high degree of chlorine substitution are generally more readily biotransformed under anoxic conditions, but are often recalcitrant to aerobic degradation (Wohlfarth and Diekert, 1997). Contaminated sites such as aquatic sediments, submerged soils, and groundwater are oxygen depleted, therefore anaerobic bacteria that are capable of organohalide respiration have been of great importance as candidates for bioremediation (van Eekert and Schraa, 2001; Smidt and de Vos, 2004; Fennell et al., 2011). Organohalide respiration is the use of halogenated compounds as terminal electron acceptors in anaerobic respiration, and is a key process for their dehalogenation in the anoxic subsurface (Smidt and de Vos, 2004; Futagami et al., 2008; Maphosa et al., 2010b). As more compounds are added to this list of organohalide pollutants that must be removed from soils, sediments, and groundwater systems, the role of ecogenomics in the elucidation of key microorganisms and consortia involved in organohalide respiration remains crucial. Finding suitable clean-up techniques for this growing range of compounds remains challenging but the more we can know about an ecosystem’s microbial potential for dehalogenation the better we can design treatment strategies and follow the processes.

## TAPPING INTO THE UNEXPLORED RESOURCES OF THE MICROBIAL HALOGEN CYCLE

**FIGURE 1:**
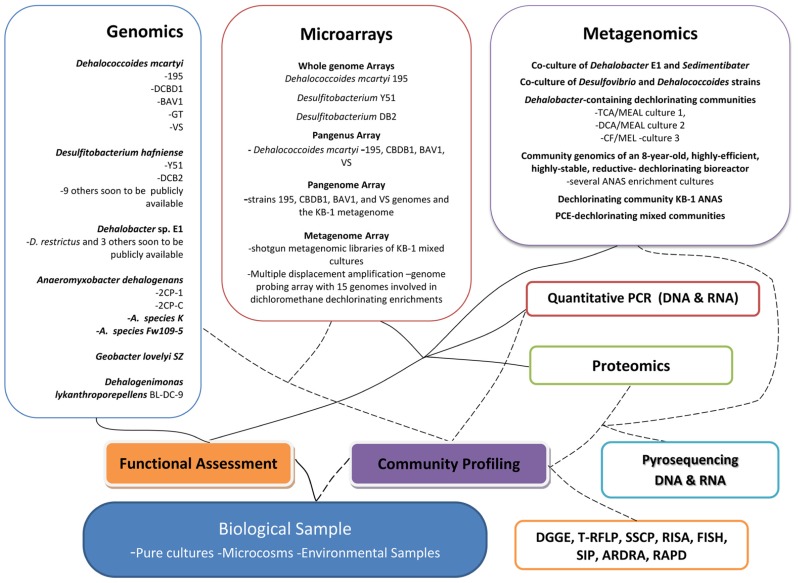
**Overview of the microbial ecogenomics toolbox-highlighting key some results and developments gained or obtained by techniques.** DGGE, denaturing gradient gel electrophoresis; T-RFLP, terminal restriction fragment length polymorphism; SSCP, single strand conformation polymorphism; RISA, ribosomal intergenic spacer analysis; SIP, stable isotope probing; ARDRA, amplified ribosomal DNA restriction analysis; RAPD, randomly amplified polymorphic DNA analysis.

Fortunately the microbial world is characterized by a vast and largely unexplored phylogenetic and functional diversity. Microbes are not only key to global biogeochemical cycles, including that of halogens, but also essential for the clean-up of polluted environments. Research over the past decade has provided considerable knowledge on a broad variety of halogenating enzyme activities, including, but not restricted to, haloperoxidases and flavin-dependent halogenases (van Pee et al., 2006). High-throughput screening for halogenase and dehalogenase activity, however, is still problematic since detection of halogenated metabolites often requires analytical chemistry solutions that are in most cases not amenable to high-throughput techniques. Additionally, the exploration of enzyme activities of reductive dehalogenases from organohalide respiring bacteria that are pivotal for the efficient clean-up of polluted subsurface environments remains challenging, because the corresponding organisms are often recalcitrant to cultivation and are restricted to anoxic environments (Smidt and de Vos, 2004). However, the emergence of ecogenomics techniques has enabled progress in addressing these challenges and has opened up the microbial “blackbox” at contaminated sites (Maphosa et al., 2010b). Recently, next generation sequencing has made it possible and financially feasible to study the metagenome of complex environmental samples harboring microbial consortia. This has not only yielded information on biodiversity, but also on putative meta-functionality of consortia present in environmental samples. To expedite the complete remediation of sites contaminated with organohalides such as chlorinated ethenes, further understanding of the physiology, biochemistry, phylogeny, and ecology of organohalide respiring consortia is warranted. Although laboratory studies give clues to *in situ* situations, often what happens in the field is different from what is observed in controlled experiments. Ecogenomics has aided in closing this gap and providing insights into the *in situ *microbial structures and functioning thus enhancing the field applications of bioremediation technologies. The ability to characterize large numbers of microorganisms from the environment, by combining phylogenetic, genomic, and biochemical analyses is crucial to developing bioremediation strategies at sites. Sadly there are gross limitations in our ability to survey microbial composition and function due to relatively poor capacity for growth of most microorganisms under *ex situ* conditions, even when using the most sophisticated resources available for culturing (Ingham et al., 2007). In order to circumvent this problem, the ecogenomics toolbox, a suite of approaches and techniques, has been developed to study communities through the analysis of their genetic material without culturing individual organisms (Handelsman, 2004; Guazzaroni et al., 2010; Maphosa et al., 2010b). The toolbox includes techniques such as quantitative polymerase chain reaction (PCR), fluorescent *in situ* hybridization, enzyme activity profiles, compound-specific isotope analysis, transcriptomics, proteomics and more recently high-throughput sequencing of (meta)genomic DNA or PCR amplified DNA from environmental samples. These tools have been used in various combinations to provide more holistic insights of microbial processes involved in organohalide degradation. This review looks at the role played by microbial ecogenomics technologies, mainly focusing on metagenomics, transcriptomics, and proteomics, to study and follow microbial community structures and functions at sites contaminated with chlorinated compounds and discusses how these findings have continued to influence the development of the monitoring toolbox and other technologies needed for polluted site clean-up (**Figure 1**).

## GAINING GLOBAL INSIGHTS ON DECHLORINATING COMMUNITIES USING METAGENOMICS

Metagenomics applications have been instrumental in providing a broad view of the genetic composition of a microbial community, including information about the identity and potential metabolic capabilities of community members. Metagenomic approaches have been used to study microbial communities associated with a wide variety of environments, for example, the termite gut, the human intestinal tract, wastewater treatment bioreactors, and acid mine drainages (Tyson et al., 2004; Gill et al., 2006; Warnecke et al., 2007; Sanapareddy et al., 2009). Having an appropriate community structure is currently understood to play a critical role in the success or failure of achieving complete dehalogenation in bioremediation systems. So far considerable study has taken place regarding the diversity of organohalide respiring bacteria and their metabolic repertoires, including the flow of energy within anaerobic communities to the microorganisms capable of rapidly producing ethene from chlorinated ethenes as probably the most widespread organohalide contaminants (Yang and McCarty, 1998; Becker et al., 2005; Heimann et al., 2006; Daprato et al., 2007). Microbial communities necessary for bioremediation purposes most often rely on intricate multispecies interactive networks. Organohalide respiring bacteria have been found to thrive in consortia, but have proven difficult to obtain in pure culture, reinforcing the need to characterize these mixed communities by cultivation-independent approaches such as metagenome sequencing. Metagenomic data can provide insights into the consortia members that support dechlorination activities (Maphosa et al., 2010b; Ding and He, 2012; Waller et al., 2012). Presently, metagenomes of defined mixed cultures and complex site-specific microbial communities are currently being unraveled, allowing determination of metabolic and sensory interactions within consortia. Several commercial dechlorinating bioreactor communities are commonly used for bioaugmentation in bioremediation systems, and their metagenomes will allow for modeling and optimization of their growth conditions at specific sites, for example, through supplying limiting nutrients and/or adjusting other environmental operating parameters.

Sometimes the individual members of the microbial community may not be easily cultured as pure strains or if cultured may not exhibit certain functions in isolation, limiting the possibility to study them individually. Taking a metagenomic approach, whereby the whole microbial community is sequenced and then partial individual genomes are teased out could help circumvent the above mentioned challenges. An example is metagenome sequencing of a defined beta-hexachlorocyclohexane dehalogenating *Dehalobacter–Sedimentibacter* coculture (Maphosa et al., 2012). The *Dehalobacter* sp. cannot be cultivated in pure culture and needs the *Sedimentibacter* sp. to maintain its ability to dechlorinate (van Doesburg et al., 2005). The draft genomes of the *Dehalobacter* and *Sedimentibacter* strains (Maphosa et al., 2012) allowed insight into the partnership dedicated to organohalide respiration, and provide an opportunity to investigate complementary gene expression to elucidate the mechanistic basis of the syntrophy between these two bacteria. The metagenome revealed that *Dehalobacter* has a greater number of reductive dehalogenases than initially anticipated. This is interesting for future exploitation of these dehalogenators since more strains and species of *Dehalobacter* are now being described that reductively dechlorinate other compounds such as chloroform and chloro- ethanes and methanes and other halogenated compounds (Grostern et al., 2009; Grostern and Edwards, 2009; Yoshida et al., 2009; Nelson et al., 2011). The presence of multiple sets of dehalogenase-encoding genes suggests that the substrate range of the *Dehalobacter* may be far greater than previously believed. *Sedimentibacter* plays a supporting function for organohalide respiration, and access to this genome sequence shows its potential role in providing, for example, cofactors for *Dehalobacter*.

Until recently research mainly focused on elucidating the identity and catabolic capacity of the dechlorinating bacteria in mixed communities. However, since recently more emphasis is being given to understanding the variations of fermentative and other supporting community members and their effects on dechlorination activity (Freeborn et al., 2005; Daprato et al., 2007; Lee et al., 2011). In these complex systems the organohalide respiring members may make up only a small percentage of the total microbial community. Thus, it is important to understand the structure and function of the rest of the microbial community and what impact they might have on bioremediation processes. Using molecular fingerprinting techniques such as DGGE, and TRFLP profiling of PCR-amplified 16S ribosomal RNA gene fragments to study anaerobic dechlorinating communities showed that while complete dechlorination of PCE-to-ethene occurred in different microcosms, the dechlorinating populations were similar, but not identical and used different fermentation pathways, with or without methanogenesis (Daprato et al., 2007). Gaining an understanding of the total community and its interaction can help to answer such questions as to why there is delayed ethene production often observed at field sites although the appropriate *Dehalococcoides *organisms are present (Daprato et al., 2007). Metagenomics in combination with (meta)transcriptomics and proteomic approaches, the latter of which focusing on actual microbial activity at a given sampling point in time and space, will be able to address these issues.

An example has been the recent metagenome sequencing of the anaerobic microbial consortium enriched from contaminated sediments taken from Alameda Naval Air Station (ANAS) in California that dechlorinates TCE to ethene (Richardson et al., 2002; Freeborn et al., 2005; Holmes et al., 2006). Through metagenome data the specific *Dehalococcoides* DNA sequences and those of the other ANAS community members were identified and examined. Elucidation of the community structure was obtained showing that phylogenetic composition of ANAS described by metagenomic sequencing generally confirmed the composition described by previous 16S rRNA gene clone library and phylogenetic microarray studies (Brisson et al., 2012). Beyond the two populations of *Dehalococcoides*, the authors also identified low G + C Gram-positives (mostly *Clostridium *and *Eubacterium *sp.), *Bacteroides* sp., *Citrobacter* sp., and *δ-Proteobacteria* (mostly *Desulfovibrio* sp.; Richardson et al., 2002; Freeborn et al., 2005). The metagenome sequence data showed variations in the relative abundances of some taxa and possible variability in the methanogen population when compared to what had been shown by the other molecular tools (Brisson et al., 2012). Metagenomic analysis has provided some insight into the functions and interactions of different community members in the context of overall TCE dechlorination activity. Firstly, *Dehalococcoides *appear to be the dominant reductive dechlorinators in ANAS. The reductive dehalogenase genes identified were all most closely related with those found in genomes of different strains of *Dehalococcoides mccartyi *(formerly* Dehalococcoides *spp.; Loffler et al., 2012). Secondly, abundant hydrogenase genes were found highlighting the importance of hydrogen metabolism in this community. The phylogeny of the ANAS community suggests there are developed working syntrophic relationships, between the different hydrogen producers (fermenters, homoacetogens) and consumers (reductive dechlorinators and methanogens). Despite known differences in thermodynamic requirements and hydrogen thresholds for the dechlorinators and methanogens (Fennell and Gossett, 1998; Löffler et al., 1999), stable long-term dechlorination activity was still achieved in this consortium containing competitive hydrogen consumers. Thirdly, important insight was gained regarding the genes related to synthesis of cobalamin, an important cofactor for reductive dechlorination. These genes were present in several community members, including *Dehalococcoides*, suggesting a unique adaptation of the ANAS strains to reductive dechlorination, but also suggesting that the non-*Dehalococcoides *community members likely have additional important roles beyond cobalamin biosynthesis (Brisson et al., 2012). Understanding the interactions within this community could enable manipulation of the various site conditions toward better optimization of the use of such consortia in bioremediation.

Still more metagenome sequencing projects are currently ongoing and should give a wealth of information for optimizing microbial function for bioremediation of organohalide-contaminated sites. Sequencing these metagenomes will not only provide more information on how the microbial community performs the dechlorination process, but could also aid in identifying other potential contaminants that these microbes can break down. Additionally, the information will allow researchers to do comparative analyses between organohalide respiring bacteria such as *Dehalococcoides*, *Dehalobacter*, and *Desulfitobacterium* but also different dechlorinating consortia to better understand these microbes, their particular metabolic processes and interactions in consortia. Metagenomics allows us to study the context of microbial community dynamics and interrelationships, including nutrient exchange and/or supply between different phylotypes in addition to the roles of specific organohalide respiring bacteria involved in breaking down chlorinated pollutants. Additionally, some of the microbial community members involved with organohalide respiring bacteria such as hydrogen producers and methanogens are of interest to sequence as they can give information useful for other purposes such as bioenergy production. Further, production of excessive methane in aquifers may be undesirable from a safety perspective. Thus, understanding methanogenic activity during bioremediation when an electron donor must be added to stimulate organohalide respiration is critical. Thus, a broader understanding of the microbial community may aid biotechnological manipulation to lessen energy demands and costs in bioremediation setups.

## HIGH-THROUGHPUT PROFILING OF COMMUNITY STRUCTURES AND FUNCTION

High-density phylogenetic microarrays, mostly targeting 16S rRNA genes (Zhou, 2003; Bodrossy and Sessitsch, 2004; DeSantis et al., 2007) can provide insights into the *in situ* microbial ecology and population dynamics at contaminated field sites undergoing bioremediation. They can be used to study changes in dechlorinating communities. For example, the PhyloChip was applied to track bacterial and archaeal communities through different phases of remediation at Ft. Lewis, WA, a trichloroethene (TCE)-contaminated groundwater site (Lee et al., 2012b). The analysis revealed that the bacterial communities were constantly changing during the course of the study. Additionally, the archaeal community showed significant increases in methanogens at the later stages of treatment that correlated with increases in methane concentrations of over two orders of magnitude (Lee et al., 2012b). Information about such changes can be crucial for maintenance of dechlorination activities at sites. The diversity captured by the PhyloChip is also more comprehensive than reported for 16S rRNA clone libraries (Bowman et al., 2006; Lee et al., 2012b), and can therefore be an effective way also to monitor the changes in abundant bacterial phyla that can influence the activity of the dechlorinators. Continued use of these chips will also lead to new clues for phyla or subfamilies that may need further investigation based on observed associations within dechlorinating communities.

In another study using carbon isotope fractionation changes to analyze degradation within a plume it was found that the *δ*13C of the CH_4_ increased from -56‰ in the source area to -13‰ with distance from the injection well, whereas the *δ*13C of dissolved inorganic carbon decreased from 8‰ to -13‰. These changes were indicative of changes in microbial activities shifting from methanogenesis to methane oxidation. This was confirmed by PhyloChip microarray analyses of 16S rRNA genes obtained from the groundwater microbial communities. There were decreasing abundances of reductive dechlorinating microorganisms such as *Dehalococcoides* and increasing CH_4_-oxidizing microorganisms capable of aerobic co-metabolism of TCE such as *Methylosinus trichosporium* along the plume axis (Conrad et al., 2010). Follow-up microcosm studies showed that electron donor amendment designed to stimulate reductive dechlorination of TCE may also stimulate co-metabolism of TCE (Conrad et al., 2010). However, it should be noted that in most applications the PhyloChip is used to detect 16S rRNA gene fragments amplified from the samples and hence like all DNA-based analyses only indicates that the organisms were present in the groundwater but not necessarily active at the time of sampling. It is possible that microbes transported from up-gradient sites or from mixing of groundwater from different depths within the wells will be detected, leading to mixing of microbes from zones of different activity. Nevertheless, useful information is obtained along different monitoring gradients (such as depth, distance, and time) providing indication to microbial structures and potential functionalities.

Pyrosequencing of PCR-amplified 16S rRNA genes has improved the resolution high-throughput profiling of microbial community analysis. For example, pyrosequencing of biofilm communities impacted by mixtures of chlorinated solvents (TCE, trichloroethane, and chloroform), revealed significant microbial community shifts related to the input of the chlorinated solvents and the onset of sulfate reduction (Zhang et al., 2010). Input to the biofilm of a mixture of three chlorinated solvents coincided with the onset of sulfate reduction and led to a more diverse community that included sulfate-reducing bacteria (*Desulfovibrio*) and nitrate-reducing bacteria (*Geothrix* and *Pseudomonas*). Interestingly, the relative abundance of *Dehalococcoides* increased in response to the addition of a low concentration of TCE as a single chlorinated solvent, but decreased when a mixture of chlorinated solvents was fed to the biofilm (Zhang et al., 2010). Such information can be crucial for developing bioremediation processes at sites with mixed chlorinated contaminants.

Recently, pyrosequencing of bacterial 16S rRNA genes in combination with 16S rRNA gene-targeted qPCR was done for microcosms developed from tidal flat samples (Lee et al., 2011). The results suggest that tidal flats harbor novel, salt-tolerant dechlorinating populations, and that pyrosequencing provided more detailed insight into community structure dynamics of the dechlorinating microcosms than conventional 16S rRNA gene sequencing or fingerprinting methods. Specifically, *Desulfuromonas michiganensis*-like populations predominated in the TCE and *cis*-DCE producing microcosms and *Dehalococcoides* spp. populations were not detected in these sediments before or after incubation with PCE. Community structures also appeared to be dependent on the depth from which sediments were obtained, having *Desulfuromonas thiophila* and *Pelobacter acidigallici*-like populations in the surface sediment microcosms, and *Desulfovibrio dechloracetivorans* and *Fusibacter paucivorans*-like populations in the deeper sediment microcosms (Lee et al., 2011). An understanding of microbial community changes along certain gradients can be crucial for engineering bioremediation interventions. Additionally, comparison of the pyrosequencing data to clone library analysis showed that the community structures were very similar suggesting the validity of pyrosequencing method and its robustness for bacterial community analysis. Secondly, the expected advantage of high output next generation sequencing approaches such as pyrosequencing towards the detection of rare populations not detected by Sanger sequencing was shown (Zhang et al., 2010; Lee et al., 2011; Shokralla et al., 2012).

To get insight into microbial processes at contaminated sites, the GeoChip can be applied (He et al., 2007, 2010). It targets functional genes for microbially mediated biogeochemical processes, such as C, N, and S cycling, P utilization, organic contaminant degradation, and metal resistance and reduction. Numerous studies have demonstrated that the GeoChip is a powerful tool and holds great promise for addressing fundamental questions relevant to global climate change, bioremediation, bioenergy, agricultural operations, land use, ecosystem restoration, and human health, and for linking microbial structure to geochemical processes and ecosystem functioning (He et al., 2011). The GeoChip has also been used for identification of key functional genes in different organohalide respiring consortia, along with other geochemically important bacterial processes (Tas et al., 2009). Amended with probes targeting 153 reductive dehalogenase genes, the GeoChip 2.0 was used to analyze microbial communities from two pesticide-impacted European rivers, the Ebro in Spain and the Elbe in Germany. It was shown that there were spatial and temporal fluctuations in the abundance of *Dehalococcoides* spp., composition of other organohalide respiring bacteria populations, as well as the reductive dehalogenase gene diversity. For example, samples from locations which had high hexachlorobenzene concentrations, were dominated by reductive dehalogenase genes of *Dehalococcoides mccartyi* CBDB1 and *D. mccartyi* 195, while samples taken from a location with a broader range of contaminants had a wide spectrum of reductive dehalogenase genes including those from various other organohalide respiring bacteria (Taş, 2009). This analysis provided insight into the natural occurrence and dynamics of active *Dehalococcoides* populations in the pesticide-contaminated river basins. Additionally, the impact of the C/N ratio, depth, total N, and location as drivers for determining the ecosystem structure and function at these sites was observed (Taş, 2009). Geochips have also been used to study sites contaminated with PAH s (Zhang et al., 2012) and polychlorinated benzenes (Leigh et al., 2007). The findings from these studies improve our understanding of pollutant degradation and carbon flow in soil and rhizospheres, and may benefit bio- and (phyto-) remediation research by facilitating the development of molecular tools to specifically detect, quantify and monitor populations and genes involved in degradative processes.

## LINKING SPECIFIC GENE EXPRESSION TO ORGANOHALIDE POLLUTANTS/STRESS

The ecogenomics toolbox has been used for profiling the functional gene expression and corresponding proteins known to be involved in organohalide respiration and also for understanding the overall responses of these organohalide respiring bacteria to other anthropogenic impacts such as nutrient availability, toxic organic compounds, and presence of heavy metals. Understanding the active functions of the microbial community aids the development and monitoring bioremediation of chlorinated sites by providing identification of microbial indicators for chlorinated compound degradation and general ecosystem health.

The application of microarrays to query the genomes of pure and mixed cultures has provided an in-depth understanding of putative gene functions not only of targeted organisms, for which the array was designed, but also of related but non-sequenced strains. Microarray technology has successfully been applied in recent years in comparative genomic analyses of related strains of bacteria in a variety of genera (Poly et al., 2004; Witney et al., 2005; Cooke et al., 2008; Boesten et al., 2009; Klaassens et al., 2009), including *Dehalococcoides* (West et al., 2008; Lee et al., 2012a) and *Desulfitobacterium* (Kim et al., 2012; Peng et al., 2012).

A whole genome array was used to further understand the physiology of *Desulfitobacterium hafniense* Y51, in order to optimize its use as a bioremediation agent at PCE-contaminated sites, and also improve on the designing of bioarray sensors to monitor the presence of dechlorinating organisms in the environment. Genome content and parallel physiological studies and transcriptomics analysis support the cell’s ability to fix N_2_ and CO_2_, form spores and biofilms, reduce metals, and use a variety of electron acceptors in respiration, including a wide range of organohalides (Peng et al., 2012). Global transcriptome analyses were performed using various electron donor and acceptor couples (respectively, pyruvate and either fumarate, TCE, nitrate, or DMSO, and vanillate/fumarate), that are known to sustain growth of strain Y51 (Peng et al., 2012). Transcriptomic data provided an initial view of the very complex physiology of strain Y51, given its ability to survive in various environmental conditions with or without PCE. During degradation of TCE as terminal electron acceptor, a series of electron carriers comprising a cytochrome bd-type quinol oxidase, a ferredoxin and four Fe–S proteins are up-regulated, suggesting that the products of these genes are involved in PCE oxidoreduction (Peng et al., 2012). Knowledge of the electron flow within this, and other organohalide respiring bacteria, is important for determining biostimulation substrates.

Microarray studies involving a *Dehalococcoides* whole genome array have been used to monitor dynamic changes in the transcriptome of *Dehalococcoides mccartyi* strain 195 (former *Dehalococcoides ethenogenes* strain 195) along a timeline from exponential to stationary phase in an effort to understand factors that limit the growth of this slow-growing isolate (Johnson et al., 2008). It was found that strain 195 can uncouple dechlorination from net growth, an important clue for optimizing use of this bacteria in bioremediation. A large number of genes located within integrated elements including a putative prophage and a multicopy transposon were up-regulated alongside genes involved in general stress response, transcription, and signal transduction while genes involved with translation and energy metabolism were down-regulated. Surprisingly and warranting further investigation was the high up-regulation of four putative reductive dehalogenases during this transition from exponential to stationary phase (Johnson et al., 2008).

The response of *Dehalococcoides* to different concentrations and forms of corrinoid cofactors has been studied with this microarray showing that strain 195 adjusts its metabolism according to the corrinoid forms available for uptake (Johnson et al., 2009). Although corrinoids are essential for dehalogenation activity, strain 195 cannot biosynthesize corrinoids *de novo* and therefore it is crucial to understand how this microorganism obtains these necessary cofactors. A pangenome model of *Dehalococcoides* suggested that evolution of *Dehalococcoides* species is driven by the electron acceptor availability (Ahsanul Islam et al., 2010). While the synthesis pathway is incomplete in *Dehalococcoides* the pangenome model suggests that *Dehalococcoides* might have evolved syntrophically with cobalamin secreters and never faced significant evolutionary pressure to acquire or maintain a complete cobalamin synthesis pathway in their genomes (Ahsanul Islam et al., 2010). Furthermore, the *Dehalococcoides mccartyi* strain 195 microarray has been used to monitor the effects of nitrogen status on *Dehalococcoides *spp. The study identified a number of genes that could potentially be used as biomarkers including the *nif *operon, ammonium transporter, PII nitrogen regulatory protein, and methylglyoxal synthase genes and a number of general stress response genes (Lee et al., 2012a). Understanding nitrogen status can help guide field scale manipulations such as addition of ammonium supplements to improve bioremediation. Additionally, using the *Dehalococcoides mccartyi* 195 microarray provided insight into *Dehalococcoides mccartyi* strain MB’s complex nutrient requirements and its restricted metabolism to organohalide respiration (Tang et al., 2009b).

Application of this array has also been used to distinguish between *Dehalococcoides* subgroups in mixed cultures (West et al., 2008). The ability to distinguish between strains from site samples is important for inference of potential functions for dechlorination. Often multiple reductive dehalogenases contributed by different dechlorinating strains are needed for complete dechlorination of chlorinated compounds. The characterized strains of *Dehalococcoides* differ in their usage of organohalides, depending on the repertoire of specific reductive dehalogenases they carry (Löffler and Edwards, 2006). As such, useful biomarkers for field diagnostic purposes for bioremediation of sites contaminated with chlorinated ethenes currently include four reductive dehalogenase encoding genes which have experimentally determined dechlorination functions (*pceA*, *tceA*, *vcrA*, and *bvcA*; Magnuson et al., 1998; Magnuson et al., 2000; Krajmalnik-Brown et al., 2004; Müller et al., 2004). Integration of the microarray results with the physiology of the cultures demonstrated that strains that are phylogenetically related on the genome level can be physiologically incongruent, suggesting that the dechlorination functions of *Dehalococcoides* cultures are independent of phylogenetic affiliation but are dictated by a small number of reductive dehalogenase-encoding genes (Lee et al., 2012b). Knowing which strains are present and the specific genes they carry is important in decision making for biostimulation or bioaugmentation at sites. For example, lack of reductive dehalogenases targeting a specific compound could suggest the need for bioaugmentation.

Transcriptomic and proteomic approaches have provided insights into the metabolism of organisms that cannot be grown in amounts sufficient to perform many standard biochemical analyses such as enzyme purification and activity measurements (Morris et al., 2006). Transcriptomic and proteomic approaches can be used to study the molecular responses of organohalide respiring bacteria to different conditions enabling the development of conceptual models to describe interactions between different cellular components and the environment. Organohalide respiring bacteria thrive within consortia that contain other anaerobes, such as *Desulfovibrio*, *Eubacterium*, *Acetobacterium*, *Citrobacter*, *Spirochetes*, and *Clostridium*, which are able to ferment organic substrates into hydrogen and acetate (Richardson et al., 2002; Duhamel and Edwards, 2006; Lee et al., 2006; Fennell et al., 2011).

For direct experimental measurement of the specific effects of associated bacteria on the growth, activity and gene expression of *Dehalococcoides*, co- and tri-cultures of *Dehalococcoides mccartyi* strain 195 with *Desulfovibrio vulgaris* Hildenborough and *Methanobacterium congolense* were studied using a combination of transcriptomics and proteomics (Men et al., 2012). The transcriptome and proteome of *Dehalococcoides* 195 grown in the co-culture showed significant differences in gene expressions and protein profiles compared with *Dehalococcoides* 195 grown in isolation. No significant transcriptomic and proteomic differences were observed between co- and tri- cultures (Men et al., 2012). Close analysis of the physiological, transcriptomic, and proteomic results indicate that the robust growth of *Dehalococcoides* 195 in co- and tri-cultures occurs because of the advantages associated with the capabilities of *Desulfovibrio vulgaris* to ferment lactate providing H_2_ and acetate for growth, along with potential benefits from proton translocation, cobalamin-salvaging and amino acid biosynthesis, whereas *Methanobacterium congolense* in the tri-culture provided no significant additional benefits beyond those of *Desulfovibrio vulgaris *(Men et al., 2012). Such an approach shows that *Dehalococcoides *can be sustained for dechlorination when appropriate syntrophic partners are present. Studies in a consortium having multiple *Dehalococcoides* have also shown that methanogens and sulfate-reducing bacteria can play a significant role in dechlorination by *Dehalococcoides* (Futagami et al., 2011). Similarly, also *Dehalobacter* sp. E1 was found to be only able to dechlorinate when grown together with a *Sedimentibacter* sp. (van Doesburg et al., 2005).

Proteomics has been useful in identifying reductive dehalogenases involved in utilization of additional substrates by, for example, *Dehalococcoides*. This has provided useful insights into reductive dehalogenase functions and their potential activities at contaminated sites. It was demonstrated that peptides from functional enzymes responsible for determining phenotypes may be used as biomarkers to differentiate closely related strains of bacteria found within the same consortia. Whereas housekeeping genes of different strains of *Dehalococcoides mccartyi* share more than 85% similarity at the amino acid level, different strains are capable of dehalogenating diverse ranges of compounds, depending on the reductive dehalogenase genes that each strain harbors and expresses (Morris et al., 2007). For example, analysis of three reductive dehalogenases, in PCE-grown cells of *Dehalococcoides mccartyi* strain 195, PceA and TceA were detected with high peptide coverage but not the DET0162 gene product. Cells grown on 2,3-dichlorophenol produced PceA with high coverage but not TceA, DET0162, or any other potential reductive dehalogenase encoded by the genome (Fung et al., 2007). Indications from proteomic studies in *Dehalococcoides* suggests that probably only low levels of reductive dehalogenases and many of the other oxidoreductases are needed to support the slow growth rates required to maintain populations in reactors (Morris et al., 2006). Such information is critical for determining which genes to target in monitoring the microbial potential and activity at contaminated sites with different chlorinated compounds.

## FROM PANGENUS TO PANGENOME AND METAGENOME ARRAYS

Sequencing and annotation of the genomes of five *Dehalococcoides mccartyi* strains (195, CBDB1, BAV1, VS, and GT) have established a reference for the genomic characteristics of this genus (McMurdie et al., 2009; Ahsanul Islam et al., 2010). Using sequences from four of these *Dehalococcoides mccartyi* genomes (195, CBDB1, BAV1, VS) a pangenus microarray using unique probe sets to target all identified genes was designed, constructed and used for comparative genomic analysis of isolates from the ANAS enrichment culture (Lee et al., 2012b). This pangenus array is well suited for the four *Dehalococcoides *genomes it represents and provides useful information for unsequenced *Dehalococcoides* genomes exhibiting a high sequence similarity to the genomes used for the design (Hug et al., 2011). Similar pangenus microarrays can be expected for *Desulfitobacterium* spp. and *Geobacter *spp. with the increasing availability of whole genome sequences. To this end, it is interesting to note that currently nine additional genomes of *Desulfitobacterium* spp., as well as those of three isolates and co-cultures of *Dehalobacter* spp., are being analyzed in the framework of the JGI community sequencing program (Kruse, unpublished data).

Since *Dehalococcoides* strains present within a community may have differing dechlorination abilities it is often necessary to identify and distinguish between them. To this end, a pangenome oligonucleotide microarray was designed based on clustered *Dehalococcoides *genes from five different sources – strain 195, CBDB1, BAV1, and VS genomes and the KB-1 metagenome (Hug et al., 2011). This pangenome probe set provides coverage of core *Dehalococcoides* genes as well as strain-specific genes while optimizing the potential for hybridization to closely related, previously unknown *Dehalococcoides* strains. It was also shown that the probe design was robust to cross-hybridization from environmental bacteria such as *Dehalogenimonas* spp. that are closely related to *Dehalococcoides* spp. Newly available *Dehalococcoides* genes from the KB-1 consortium were used to confirm the applicability of this probe set and microarray for universal detection of *Dehalococcoides*. *In silico* comparisons to the *Dehalococcoides* strain GT genome indicated that the pangenome probe set detects a larger proportion of a novel *Dehalococcoides* strain’s genes than those from the strain-specific or pangenus arrays (Hug et al., 2011). Detection and identification of *Dehalococcoides* at contaminated sites and pristine sites where organohalide respiring bacteria organisms have not yet been exposed to contamination can be done using this pangenome array. The pangenome array has the potential to become a common platform for *Dehalococcoides*-focused research, allowing meaningful comparisons between microarray experiments regardless of the strain examined, thereby enabling detailed detection and characterization of even unknown *Dehalococcoides* population structures and metabolic functions from contaminated sites (Hug et al., 2011).

Although a large fraction of dechlorinating microbial consortia is still yet uncultured it is still desirable to study their involvement in bioremediation using microarrays in order to take advantage of the high sensitivity and specificity of microarray technology. As a way to overcome the challenges of studying these uncultivated bacterial genomes, multiple displacement amplification method can be used to directly amplify the genomes from single cells (Raghunathan et al., 2005; Lasken, 2007). Multiple displacement amplification makes use of an isothermal amplification technique using random hexamer primers and bacteriophage phi29 polymerase, generate large amounts of DNA, amplifying fragment sizes longer than 10 kb, has a high level of proofreading, and lower amplification bias than other genome amplification methods (Lasken, 2009). The application of multiple displacement amplification can be coupled to microarray design for analysis of environmental samples without *a priori* knowledge of microbial diversity which is often needed for development of the DNA microarrays. To demonstrate this method a digital multiple-displacement-amplification genome-probing array was developed to monitor the dynamics of dichloromethane dechlorinating communities from different phases of enrichment status (Chang et al., 2008). Fifteen genomes from key microbes involved in dichloromethane-dechlorinating enrichment were used as microarray probes. The method allowed to monitor both cultivated strains and uncultivated microorganisms the genomes of which were amplified by digital multiple displacement amplification from the dichloromethane-dechlorinating community in the microcosm phases. Significant changes related to dechlorination and growth on dichloromethane were observed for some of the genomes from uncultured microorganisms over the time course of monitoring (Chang et al., 2008). Equipped with such techniques as pangenome and multiple-displacement-amplification genome-probing array it will be possible to get more in depth knowledge of microorganisms not yet represented in current rRNA gene libraries and even allow for monitoring microbial populations.

In order to identify genes within dechlorinating communities that are transcriptionally active and critical for ecosystem function in bioremediation, metagenomic microarrays can be used whereby important genes may be detected by identifying transcripts from arrayed short-insert libraries. Such metagenomic microarrays have been used to screen for clones that contain specific genes among a large number of clones from metagenomes libraries (Park et al., 2008; Jin et al., 2009). To study the final step in vinyl chloride dechlorination a shotgun metagenomic microarray was constructed by generating two shotgun short-insert metagenomic DNA libraries from genomic DNA from the mixed culture KB-1 (Waller et al., 2012). The PCR products were spotted on the microarray. This metagenome microarray was used to compare levels of gene expression in the *Dehalococcoides* containing microbial community KB-1 in the presence and absence of the electron acceptor vinyl chloride. Results showed that during vinyl chloride degradation *Dehalococcoides* genes involved in transcription, translation, metabolic energy generation, and amino acid and lipid metabolism and transport were overrepresented in the transcripts compared to the average *Dehalococcoides* genome. Secondly, numerous hypothetical genes from *Dehalococcoides* coding for uncharacterized proteins had high transcripts level in the absence of vinyl chloride suggesting a role in cell maintenance. Thirdly, it was found that there is a *Siphoviridae*-like *Dehalococcoides* prophage that is activated in response to starvation conditions (Waller et al., 2012). This paves way for further research to understand the role of this and other prophages and their distribution in the environment, as well corresponding implications for bioremediation with *Dehalococcoides*. Starvation and other stressful conditions (high salt concentrations, heavy metals, low nutrient concentrations) are common at contaminated sites and may cause activation of *Dehalococcoides* prophages, thus allowing increased genetic variation through phage-mediated gene transfer in and possibly beyond *Dehalococcoides*. Lastly, the importance of understanding whole community dynamics was shown from the high transcript levels observed for *Spirochaetes*, *Chloroflexi*, *Geobacter*, and methanogens (Waller et al., 2012). This demonstrates yet again the importance of non-*Dehalococcoides* organisms to this culture but also other organohalide respirers. Although shotgun metagenome microarrays are a challenging approach compared to many other technologies that are being used to study gene expression in the environment, it can be an effective high-throughput screening tool for identification of novel genes that could be interesting for follow-up studies.

## UNDERSTANDING GENETIC MOBILITY

Analysis of the *Dehalococcoides* prophages mentioned above showed their association with reductive dehalogenase genes and various other mobile genetic elements. Although there is no experimental evidence yet, it was suggested that the *tceA* gene, for example, maybe occasionally packaged with the prophage DNA into mobile viral particles (Waller et al., 2012). This could very well be, as quantitative PCR analysis of reductive dehalogenases from environmental samples has repeatedly indicated higher reductive dehalogenase gene counts as compared to 16S rRNA gene counts above the expected 1:1 ratio based on the presence of single copies of both functional and rRNA genes in *Dehalococcoides mccartyi* strains (Maphosa et al., 2010a; van der Zaan et al., 2010). Prophages often constitute main sources of variation between strains and often confer beneficial environment-specific capacities to the host strain through the genes that they contain (Canchaya et al., 2003). This indicates the importance of gene transfer in the development and spread of catabolic pathways providing microbial strains with versatile metabolic abilities.

Ecogenomics techniques have also enabled us to study horizontal transfer of genes and provide an understanding of the ecological role in contributing to the diversification of organohalide respiring bacteria and in facilitating the rapid adaptation of microbial communities to ecosystems contaminated with chlorinated compounds (Maillard et al., 2005; Futagami et al., 2008; McMurdie et al., 2009; Liang et al., 2012; see Liang et al., 2012 for a detailed review of the role of horizontal gene transfer in the breakdown of chlorinated compounds). Various reductive dehalogenase genes have been found to be associated with transmissible elements, such as transposases, resolvases, insertion sequences, integrases, and recombinases (Regeard et al., 2005; Liang et al., 2012). For example, *Dehalococcoides* genomes have a conserved core that is interrupted by two high plasticity regions near the chromosomal origin of replication (Regeard et al., 2005; McMurdie et al., 2009). In these high plasticity regions, genomic islands and strain-specific genes were identified as well as an elevated number of repeated elements such as insertion sequences, including 91 of 96 *rdhAB* genes (McMurdie et al., 2009). Additionally, the vinyl chloride reductive dehalogenases (*vcrA* and *bvcA*) found in *Dehalococcoides* appear to be horizontally acquired (McMurdie et al., 2009), have a highly unusual, low percentage (G + C) codon bias, and both are found within a low percentage (G + C) genomic island that interrupts local gene synteny relative to other *Dehalococcoides* strains (Krajmalnik-Brown et al., 2004; McMurdie et al., 2007). Further structural comparison of *Dehalococcoides* genomic and metagenomic data combined with targeted sequencing from unsequenced vinyl chloride respiring enrichment cultures, resulted in the identification of homologous mobile elements containing the vinyl chloride reductase operon, *vcrABC*, that integrates at the single-copy gene *ssrA* (McMurdie et al., 2011). Similar co-localization of *rdhAB* genes with genomic islands and other signatures for horizontal transfer, have been observed in *Desulfitobacterium* and *Dehalobacter* genomes (Nonaka et al., 2006; Villemur et al., 2006; Kim et al., 2010; Maphosa et al., 2012), suggesting that niche adaptation via organohalide respiration is a fundamental ecological strategy in organohalide respiring bacteria.

The tetrachloroethene reductive dehalogenase operon *pceABCT* in *Desulfitobacterium* and *Dehalobacter* genomes is flanked by a composite transposon structure which has insertion sequences (IS) belonging to the IS*256* family (Maillard et al., 2005). In *Desulfitobacterium hafniense* strain TCE1, indirect indications for circular insertion sequences and transposon intermediates have been observed, suggesting that there is an active transposition (Maillard et al., 2005). Furthermore, genetic rearrangements were also observed to be occurring around the *pce* gene cluster in *Desulfitobacterium* hafniense strain TCE1 in the absence of PCE. This highlights the opportunistic nature of *Desulfitobacterium* toward organohalide respiration with PCE, as this capability was rapidly lost when not required (Duret et al., 2012). A similar phenomenon of possible reductive dehalogenase gene loss was also described for *Sulfurospirillum multivorans* after long term cultivation in the absence of chlorinated compounds (John et al., 2009). It is suggested that the drastic elimination of *pce* genes or loss of transcription indicates that *Desulfitobacterium* spp. have not yet fully evolved towards a dedicated metabolism of organohalide respiration and that *Desulfitobacterium* isolates harboring the *pce* transposon could have acquired it by horizontal gene transfer from obligate organohalide respiring bacteria such as *Dehalobacter* (Duret et al., 2012). Horizontal gene transfer events may therefore influence the physiology and signal responses involved when a given microbial community is facing a contaminated environment.

*Geobacter lovleyi* is a unique member of the Geobacteraceae because strains of this species share the ability for organohalide respiration. Comparative genome analysis of *G. lovleyi* identified genetic elements associated with organohalide respiration and elucidated genome features that distinguish *G. lovleyi* strain SZ, for example, from other members of the *Geobacteraceae* (Wagner et al., 2012). Strain SZ’s expanded respiratory capabilities toward organohalides is due to gene acquisitions resulting in the reductive dehalogenases being located on chromosomal genomic islands but also through lateral acquisition of a plasmid replicon (Wagner et al., 2012). Systems biology approaches combining functional genomics, metagenomics, transcriptomics, and proteomics of organohalide respiring bacteria and communities should be employed not only to reveal the detailed mechanism of horizontal gene transfer but also to fully understand how gene mobility impacts bioremediation processes at large.

## FUTURE PERSPECTIVES AND CONCLUSION

By using data from advanced molecular tools such as metagenomics, tag-pyrosequencing, transcriptomics, and proteomics it now becomes possible to obtain a comprehensive time- and space-resolved view of the subsurface microbial community structures and functions. Equipped with the new data, complementary qPCR analysis targeting specific key functional biomarkers can be better designed for routine monitoring and site analysis (Lee et al., 2012a). For example, *Dehalococcoides* and reductive dehalogenases (*pceA*, *tceA*, *bvcA*, and *vcrA*) are now commonly used key biomarkers for determining potential or activity at sites contaminated with chloroethenes. However, still more specific knowledge is needed for better implementation and use of these biomarkers and possibly identification of better markers. For example, the production of vinyl chloride by these enzymes (PceA and TceA) maybe even more harmful than corrective so a more comprehensive knowledge of the enzymes present at a site is therefore crucial to determining the extent and direction of the invention in order to prevent worsening the situation. From the increasing knowledge obtained thus far *Dehalobacter* spp. are now also emerging as potential biomarkers for sites contaminated with chloroethanes, and this will likely develop as more reductive dehalogenases are identified and characterized from this phylum (Scheutz et al., 2011).

From these transcriptomics and proteomic studies we now beginning to understand that reductive dehalogenase gene expression may not always correlate with dechlorination activity and that up-regulation of reductive dehalogenases is actually a stress response. Future studies are needed to overcome misleading results obtained from the analysis of *Dehalococcoides* transcripts which may lead to erroneous conclusions for example at sites undergoing thermal treatment (Fletcher et al., 2011) or in oxygen-stressed environments (Amos et al., 2008). Challenges that still need to be addressed to improve performance of microarray technology for reliable routine analysis of field samples include: (i) optimization of coverage of probe sets to be better able to track genes in environmental samples, (ii) concentrations of DNA and RNA needed for the analysis, and (iii) standardization of hybridization protocols including data analysis and interpretation. Genomic and proteomic databases need proper curation in order to allow for consistent extrapolation of peptide data gained from proteomic analysis.

Beyond genomes and proteomes another quickly developing area is the study of metabolites – *metabolomics*. Metabolomics seeks to detect amino acids, nucleosides, nucleotides, organic acids, redox cofactors, and the metabolic intermediates of various cellular processes and tries to understand the dynamic changes and fluxes in (microbial) cells. Metabolomics will allow us to assess gene function and relationships to phenotypes, understand metabolism and predict novel pathways, assess effects of genetic and metabolic engineering, and gage the effect of environment stress changes that lead to changes in gene expression and metabolite levels (Burja et al., 2003; Singh, 2006). Several studies have recently applied microbial metabolome analysis to study biodegradation of anthropogenic pollutants such as degradation of phenanthrene by *Sinorhizobium* sp. C4 (Keum et al., 2008), co-metabolic pathways for bioremediation of toxic metals, radionuclides, and organohalides in *Shewanella* sp. (Tang et al., 2009a). These studies have shown that metabolism is regulated at genomic, transcriptional, and posttranslational levels, and that fluxes of cellular molecules/metabolites within a cell over a time period (fluxomics) are important in understanding ecosystem function (Wiechert et al., 2007; Desai et al., 2010). Similar system-wide approaches combining genomics and proteomics with metabolomics would certainly improve our understanding and prediction of the activities of organohalide respiring bacteria at sites contaminated with chloroethenes (Villas-Boas and Bruheim, 2007; Mapelli et al., 2008). Metabolomics techniques provides a more holistic approach by analyzing as many metabolites as possible, enabling detailed analysis of substrates and degradation intermediates as well as all intracellular metabolites (Villas-Boas and Bruheim, 2007). This will give comprehensive descriptions of microbial catabolic pathways greatly facilitating the improvement of degradation processes *via* pathway-engineering and the implementation of effective bioremediation strategies *in situ*.

New techniques will be needed to allow for assessing the viable versus non-viable microbial component at contaminated sites. For example, building on protocols established in medical and food microbiology to quantitatively distinguish viable and non-viable (dead) cells by pretreating samples with ethidium monoazide or propidium monoazide before DNA extraction and qPCR analysis (Li and Wackett, 1992; Schanke and Wackett, 1992; Wackett, 1992; Amos et al., 2008; Li et al., 2011). Similar protocols can be implemented in analysis of bioremediation sites (Amos et al., 2008). As rapid developments of these techniques continue, challenges have to be overcome concerning biases observed on microarrays and limitations of sequence databases.

High-throughput culturing techniques such as the MicroDish Culture Chip platform (Ingham et al., 2007) coupled with the ecogenomics toolbox will enable us to tap into and gain insight on the yet uncultured microbial biodiversity involved in organohalide respiration. In most molecular microbial ecological studies that have been published in recent years cultivation of detected abundant microorganisms is rarely considered. The function of microorganisms in the environments is often deduced from the physiological properties of the closest cultured relatives, but often the predicted function is questionable at best. In such cases, advanced functional (meta)genomics-based approaches can be applied to study and predict the function of microorganisms in environmental processes. Such approaches include cloning and sequencing of rRNA genes and functional genes (Roest et al., 2005; Ariesyady et al., 2007; Klocke et al., 2007; Sousa et al., 2009; Pereyra et al., 2010); microautography combined with fluorescent *in situ* hybridization (FISH-MAR; Rossello-Mora et al., 2003; Wagner et al., 2006), stable isotope probing (SIP; Lueders et al., 2004; Hatamoto et al., 2007; Kovatcheva-Datchary et al., 2009), and nano-secondary ion mass spectrometry (Nano-SIMS; Behrens et al., 2008; Wagner, 2009). In addition, meta-genomic approaches are used to unravel the structure and function relationships in microbial communities (Schlüter et al., 2008; Szczepanowski et al., 2008; Krober et al., 2009; Qin et al., 2010; Zhou et al., 2010). These methods can be used to detect the presence and activity of specific microorganisms in the environment, however, detailed physiological and biochemical studies are only possible when the microorganisms have been cultured and isolated. Therefore, beyond the use of ecogenomics techniques, research using novel isolation and screening technologies followed by into-depth characterization studies remains essential to obtain insight into the role of particular microorganisms in geochemical cycles and to explore their potential for biotechnological purposes at sites contaminated with organohalides.

With these ecogenomics tools scientists are in a better position to answer questions such as how oxygen stress, nutrient availability, or high contaminant concentrations along differing geochemical gradients or at transitional interfaces impact the organohalide respiring community structure and function. Ultimately, by tracking the overall microbial community structure and function in addition to key functional players, informed decisions can then be made regarding how to best manipulate the field conditions to achieve effective bioremediation of, e.g., chlorinated ethenes.

## Conflict of Interest Statement

The authors declare that the research was conducted in the absence of any commercial or financial relationships that could be construed as a potential conflict of interest.
